# The role of recoding in implicit social cognition: Investigating the scope and interpretation of the ReAL model for the implicit association test

**DOI:** 10.1371/journal.pone.0250068

**Published:** 2021-04-20

**Authors:** Jimmy Calanchini, Franziska Meissner, Karl Christoph Klauer

**Affiliations:** 1 Department of Psychology, University of California, Riverside, California, United States of America; 2 Friedrich-Schiller-Universität Jena, Jena, Germany; 3 Department of Social Psychology and Methodology, Albert-Ludwigs-Universität Freiburg, Freiburg im Breisgau, Germany; Universita degli Studi di Milano-Bicocca, ITALY

## Abstract

The ReAL model is a multinomial processing tree model that quantifies the contribution of three qualitatively distinct processes–recoding, associations, and accuracy–to responses on the implicit association test (IAT), but has only been validated on a modified version of the IAT procedure. The initial goal of the present research was to validate an abbreviated version of the ReAL model (i.e., the Brief ReAL model) on the standard IAT procedure. Two experiments replicated previous validity evidence for the ReAL model on the modified IAT procedure, but did not produce valid parameter estimates for the Brief ReAL model on the standard IAT procedure. A third, pre-registered experiment systematically manipulated all of the task procedures that vary between the standard and modified IAT procedures–response deadline, number of trials, trial constraints–to determine the conditions under which the Brief ReAL model can be validly applied to the IAT. The Brief ReAL model estimated theoretically-interpretable parameters only under a narrow range of IAT conditions, but the ReAL model generally estimated theoretically-interpretable parameters under most IAT conditions. We discuss the application of these findings to implicit social cognition research, and their implications to social cognitive theory.

## Introduction

Implicit measures were initially designed to primarily assess mental associations stored in memory [1, 2]. However, subsequent research has revealed that responses on implicit measures reflect the influence of a variety of cognitive processes besides associations. Meissner and Rothermund [3] proposed that three qualitatively distinct processes jointly contribute to responses on the Implicit Association Test [IAT: 4]: mental associations between concepts and attributes, accuracy orientation, and a task simplification known as recoding. The specific manner in which these three processes interact to produce IAT responses was formalized as a multinomial processing tree model [MPT: 5] known as the *ReAL* model [3]. MPTs estimate the contribution of mental processes from the frequency of correct and incorrect responses; however, on the IAT, people typically make relatively few errors. Consequently, Meissner and Rothermund [3] modified the standard IAT procedure in order to increase errors by imposing response deadlines and increasing the overall number of trials, thereby increasing the reliability of parameter estimates. The ReAL model fit data from this modified IAT procedure very well and, over the course of seven experiments, Meissner and Rothermund [3] provided first evidence for the validity of parameters estimated from the modified IAT procedure. Since then, the ReAL model has been used along with modified IAT procedures to provide process-level insight on topics ranging from gender biases [6] to romantic relationships [7].

In this manuscript, we use the term ‘implicit’ to mean ‘indirect’. Thus, an ‘implicit measure’ assesses mental contents indirectly, often based on the speed or accuracy of responses, rather than on the contents of responses, per se. We use the term ‘association’ to refer to one of the mental constructs assessed by implicit measures. However, we make no strong assumptions about the representational nature of the constructs assessed by implicit measures–whether they reflect simple links between concepts [8], relatively more complex propositional structures [9], or otherwise.

The IAT is the most widely-used implicit measure. It has been used in hundreds of studies by researchers across a wide variety of domains [for reviews, see 10–12]. Since the IAT was first introduced in 1998, most IAT research has relied on a relatively standardized task procedure [4, 13]–which differs from the ReAL IAT procedure in several important ways. For linguistic convenience, throughout this article we refer to the IAT paradigm described by [13] as the ‘standard IAT procedure’, which is the paradigm currently reflected on the Project Implicit demonstration website (http://implicit.harvard.edu). We describe the IAT paradigm developed by Meissner and Rothermund [3] as the ‘ReAL IAT procedure’. Consequently, researchers interested in the cognitive processes reflected in the ReAL model would have to collect new data using the modified IAT procedure that the ReAL model has been validated on, but they may be understandably hesitant to deviate from the standard IAT procedure that has been so extensively validated, refined, and used. Moreover, to limit the ReAL model to only new-data applications would be to miss out on one of the strengths of MPTs: they can be applied post hoc to provide additional insight into existing data [14, 15]. Two barriers currently stand in the way of researchers applying the ReAL model to the standard IAT. The first barrier is that, for technical reasons that we elaborate upon below, the ReAL model will need to be reconfigured in order to be applied to a standard IAT. The second, and related, barrier is that validation evidence is always necessary when an MPT model is applied to a new task or measure, and certainly when the model is changed, in order to demonstrate that the model’s parameters reflect their intended constructs. Thus, if a version of the ReAL model can be fitted and validated on the standard IAT procedure, researchers could use it both with their familiar experimental procedures and to re-analyze existing data.

We embarked on this line of research with the goals of fitting and validating a version of the ReAL model on the standard IAT procedure. However, we quickly discovered that this would not be as straightforward an endeavor as we had initially hoped. Bem [16, p187] famously urged scientists to not write articles that reflect “a personal history of your stillborn thoughts.” Nevertheless, we believe there is scientific value in transparency, and evidentiary value in data that do not support a priori predictions when they are collected according to best practices. Consequently, in this article we report two initial experiments aimed to fit and validate a version of the ReAL model on the standard IAT paradigm, along with a third preregistered experiment designed to comprehensively identify the task procedures under which versions of the ReAL model can be validly applied to the IAT.

## The implicit association test and the role of recoding

The IAT [4] was developed to indirectly measure attitudes, which are operationalized as associations between target concepts (e.g., flowers) and attributes (e.g., pleasant). In the IAT, different stimuli appear successively onscreen, and participants are instructed to respond to them via key press according to two target categories (e.g., flower, insect) and two attribute categories (e.g., pleasant, unpleasant). Because only two response keys are available, one target category and one attribute category always share a key. For example, in one block of trials on an IAT measuring attitudes towards flowers and insects, flowers and pleasant concepts might share one response key and insects and unpleasant concepts might share the other response key. In a subsequent block of trials, the assignment of the target categories is switched, such that insects and pleasant concepts share one response key and flowers and unpleasant concepts share the other response key.

Usually, participants are able to respond more quickly and accurately to one set of response pairings (e.g., flower/pleasant) than the other (e.g., insect/pleasant). Blocks of trials in which responses are facilitated are normatively referred to as *compatible* blocks, whereas block of trials in which responses are hindered are normatively referred to as *incompatible* blocks. Such a *compatibility effect* (also known as the *IAT effect*) is often interpreted as evidence that one target category (e.g., flowers) automatically activates positive evaluations and the other target category (e.g., insects) activates negatives associations. To the extent that attitudes reflect associations between objects and evaluations stored in memory [8], the magnitude of the compatibility effect is assumed to reflect the degree of relative preference for one target category over the other. This account offers an intuitive explanation of IAT results. However, despite its high face validity, extensive research has revealed that evaluative associations are not the only cognitive processes that contribute to IAT effects [17–20].

One process that has received considerable attention is *recoding*. In the context of the IAT, recoding refers to simplifying responses based on shared features between target and attribute stimuli [19–23]. For example, flowers are typically evaluated positively whereas insects are typically evaluated negatively. Thus, in compatible blocks of the flower-insect IAT in which flowers and pleasant concepts share one response key and insects and unpleasant concepts share the other response key, participants can respond to all stimuli based on valence and can ignore target category (i.e., flower, insect) information. Hence, the double categorization task in the IAT is reduced to a simple binary decision: Is the presented stimulus pleasant or unpleasant? Such simplification is not possible in incompatible blocks because insects share one response key with pleasant concepts and flowers share the other response key with unpleasant concepts. Consequently, responding in these blocks to both target and attribute stimuli based on the shared feature of valence would lead to incorrect responses for all target stimuli, so participants must attend to the dual categories of the stimuli. The asymmetry created by task simplification (i.e., recoding) in compatible but not incompatible blocks results in relatively shorter response times and fewer errors in compatible versus incompatible blocks. Therefore, IAT compatibility effects cannot be unambiguously interpreted as evidence of relatively more positive evaluations of one target category versus the other. Instead, valence could be just a feature of the stimuli used to simplify the task via recoding [20, 22, 23].

Several approaches have been proposed as means to dissociate the underlying processes in the IAT by comparing performance across different tasks or experimental conditions [18, 20, 24, 25]. However, such *task*-*dissociation* approaches that rely on different measures to assess different processes are not ideal because they necessarily confound process with task [26]. In contrast, Meissner and Rothermund [3] applied a *process-dissociation* approach in order to separately estimate the joint contributions of recoding and evaluative associations within a single IAT.

## The ReAL model

Meissner and Rothermund [3] proposed that three qualitatively distinct processes jointly contribute to responses on the IAT: recoding, mental associations between concepts and evaluations, and label-based identification. They formalized the specific manner in which these three processes interact to produce IAT responses as a multinomial processing tree (MPT) model known as the ReAL model [3]. MPTs assume that observable responses are the result of the interplay of multiple nonobservable cognitive processes. Parameters representing these processes, and the system of equations specifying the precise manner in which they jointly contribute to responses, can be depicted as a processing tree.

**Fig 1:**
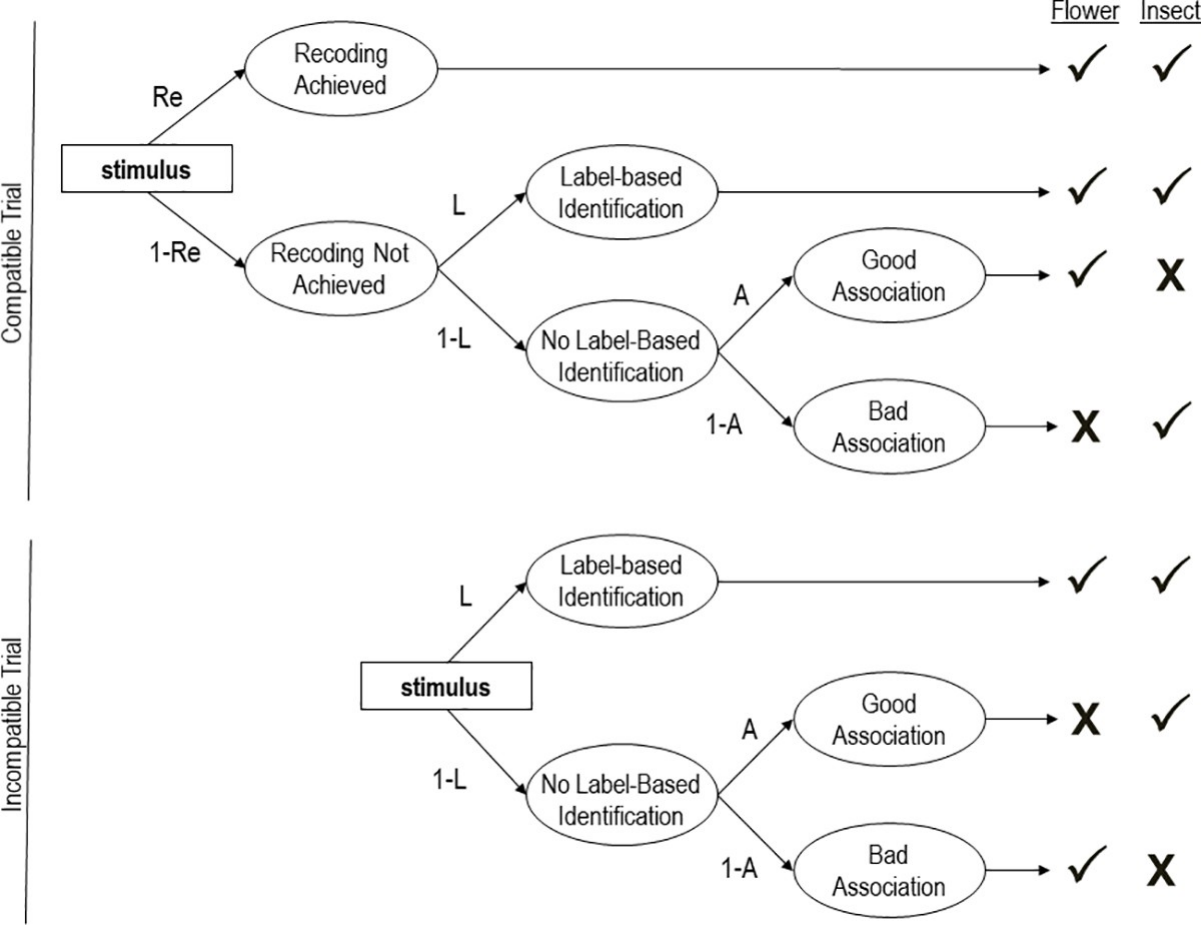
The ReAL model depicted for an IAT with flowers and insects as target categories. The figure illustrates how Recoding (*Re)*, evaluative associations (*A*), and label-based identification (*L*) jointly produce correct (**✓**) and incorrect (**X**) responses on compatible and incompatible trials.

Fig 1 illustrates the interplay of the three parameters specified in the ReAL model as a processing tree: recoding (*Re*), evaluative associations (*A*) and label-based identification of the correct response (*L*). Each path in the tree represents a likelihood, and parameters with lines leading to them are conditional on all preceding parameters. Using the flower-insect IAT as an example, there are three ways in which a correct response can be made to a flower stimulus in the compatible block, in which flowers and pleasant concepts share a response key. The first way is for the task to be simplified through recoding, such that the recoded response category that combines flowers and pleasant concepts drives the correct response, which is represented by the probability *Re*. The second way in which a correct response can be made is through the controlled search for the category label to which the stimulus belongs (e.g., flower) and for the response key that is associated with this category, which is represented by the probability *L*. Label-based identification can only drive responses when recoding does not, so this path is represented by the equation (1-*Re*) × *L*. The third way is for neither recoding (1-*Re*) nor the label-based identification process (1-*L*) to drive responses, in which case evaluative associations between flowers and pleasant concepts *A* drive the correct response, which is represented by the equation (1-*R*e) × (1-*L*) × *A*. As such, the overall likelihood of producing a correct response on a compatible trial is represented as the sum of these three conditional probabilities: *Re* + (1-*Re*) × *L* + (1-*Re*) × (1-*L*) × *A*. The respective equations for each item category (e.g., flowers, insects, pleasant words, and unpleasant words in both block types) are then used to predict the observed proportions of correct responses and errors in a given data set, and the parameter values are interpreted as relative levels of the processes. The full set of equations for the ReAL model is available in the S1 File.

Over the course of seven experiments, Meissner and Rothermund [3] demonstrated the stochastic, conceptual, discriminant, and predictive validity of the parameters in the ReAL model. The *Re* parameter is conceptualized to reflect the influence of recoding, and is diminished under conditions which minimize the opportunity to simplify the task into a binary categorization [3: Experiment 3]. The *A* parameter is conceptualized to reflect evaluative associations activated by the stimuli; it is responsive to information about novel groups [3: Experiment 4], reflects evaluations of existing groups [3: Experiments 5], and can predict behavior [3: Experiment 7]. The *L* parameter is conceptualized to reflect label-based identification of the correct response, and is sensitive to the difficulty with which stimuli can be identified [3: Experiment 2].

## Standard versus ReAL IAT procedures

Importantly, Meissner and Rothermund [3] did not validate the ReAL model using the standard IAT procedure. MPTs such as the ReAL model estimate the contribution of mental processes based on the frequency of correct and incorrect responses, but people typically make relatively few errors in the standard IAT. In order to increase errors and, thus, increase the reliability of parameter estimates [27], Meissner and Rothermund [3] developed a modified IAT procedure that imposes short response deadlines and more than doubles the overall number of trials relative to the standard IAT. Additionally, they imposed constraints on the order in which the categories of presented stimuli were repeated versus switched, such that task repetitions (e.g., two flower stimuli presented consecutively) and task switches (e.g., a flower stimulus followed by an unpleasant stimulus) occurred equally often, whereas the standard IAT switches between target and attribute stimuli on each trial. This constraint on the order in which stimuli are presented is crucial to providing the degrees of freedom necessary to estimate all of the model’s parameters, and the ReAL model fit data from this modified IAT very well.

Though Meissner and Rothermund [3] used the same, modified IAT procedure across all seven validation experiments, the ReAL model has also been applied to IATs with procedural characteristics that differ slightly from those used in Meissner and Rothermund [3], including IATs with adaptive response deadlines [3, 7, 28, Experiment 1], fixed response deadlines [6, 28, Experiment 2], and procedures that omit response deadlines altogether [29]. These findings suggest that the validity of the ReAL model is not limited to a specific operationalization of response deadline in the IAT. However, the presence or absence of a response deadline is only one of the three features which vary between the standard IAT and the ReAL IAT. To date, the ReAL model has only been applied to data derived from IATs with an extended number of trials, and with task-switch and task-repeat trials. The standard IAT includes neither of these features, so the extent to which the ReAL model will estimate valid parameter from an IAT with the standard number of trials and strictly alternating classification remains an open question.

## Procedural differences reveal process-level differences

Thus far, we have primarily framed our goal of validating the ReAL model on the standard IAT procedure as offering a methodological advancement, in that the model could be applied to more data and in other research contexts. However, the question of which IAT task procedures the ReAL model can be validly applied to also reflects important theoretical considerations.

The ReAL IAT procedure imposes short response deadlines, whereas the standard IAT procedure includes no response deadline. The ReAL IAT procedure also more than doubles the number of trials relative to the standard IAT procedure. Response deadlines are often employed in order to control for speed-accuracy trade-offs [e.g., in the affective priming paradigm; 30–33]. However, deadlines do not uniformly influence the cognitive processes that contribute to responses on implicit measures: deadlines decrease the opportunity for slow but not fast processes to influence responses [34, 35]. Similarly, increasing the number of trials in a measure depletes cognitive resources, which reduces the influence of resource-dependent but not of efficient processes [3, 36]. Taken together, the short response deadlines and additional trials reflected in the ReAL IAT procedure would seem to constrain the influence of relatively slow and resource-dependent control processes to a greater degree than does the standard IAT procedure. Thus, an investigation into differences between the two IAT procedures–and how these differences affect the ReAL model–is an investigation into the role of control in the IAT. Because an MPT reflects theory instantiated as equations, the present research is an investigation into the theoretical assumptions underlying the ReAL model.

Previous research provides some insight into the extent to which control in the IAT varies as a function of task procedures. For example, the *L* parameter of the ReAL model is conceptualized to reflect an accuracy-oriented control process, and is reduced in IATs with response deadlines [3] relative to an IAT without a response deadline [29]. Similarly, the *L* parameter is affected by the difficulty of the response deadline, and is also reduced on later trials relative to earlier trials within a given IAT [3]. Meissner and Rothermund [3] did not observe similar systematic influences of response deadline or task length on the model parameters reflecting recoding and evaluative associations. However, there has been only one ReAL model study that completely omitted response deadlines [29], and none that relied on the standard IAT procedure. Hence, it remains an open question whether the assumptions underlying the ReAL model and the validity of the model parameters depend on IAT procedures that constrain control in one way or another. For example, the *Re* parameter plays a dominant role in the model, in that the *A* and *L* parameters can only drive responses when *Re* does not. Perhaps the primacy of *Re* would be challenged under conditions in which control processes can exert greater influence. Thus, investigating the task conditions under which the ReAL model can be validly applied to the IAT could potentially identify important boundary conditions of the model.

## Replication experiments

The first two experiments we report here were intended to closely replicate the original ReAL model validation experiments from Meissner and Rothermund [3], using the same materials and stimuli that have been used in previous ReAL model research [3, 28]. Here, we extend on Meissner and Rothermund [3], in that each participant in these replication experiments completed two IAT versions that were identical in content but differed in structure. One IAT reflected the standard IAT procedure, and the other reflected the ReAL IAT procedure.

Post hoc power analyses using G*Power [37] indicate that the studies reported in Meissner and Rothermund [3] were all very well-powered to detect the effects of central interest, which we defined as the effect sizes associated with the tests that corresponded to the validity goal of each study. Power ranged from 0.86 to 0.99 at α = 0.05, with average power across studies = 0.96. That said, the effects reported in Meissner and Rothermund [3] are based on model parameters calculated at the individual level using maximum likelihood, and on traditional statistical approaches, i.e., *t*-tests and ANOVAs. In contrast, in the present research we employ a hierarchical Bayesian estimation and analysis approach (described in greater detail below). Though there is no agreed-upon procedure for conducting power analyses for this type of Bayesian analysis, our approach can be expected to be more powerful than the traditional approach, given that it constrains individual-level measurement error in the group-level estimates. However, compared to the ReAL IAT procedure, the standard IAT procedure relies on fewer data points per individual and is associated with less frequent errors due to the absence of a response deadline. To the extent that the statistical advantages afforded by our hierarchical Bayesian analyses balance out the methodological limitations of the standard IAT procedure, we aimed to collect the same size samples in these two replication experiments as were reported in Meissner and Rothermund [3].

### Replication Experiment 1

This experiment conceptually replicated Meissner and Rothermund’s [3] Experiments 1 and 3 to validate the *Re* parameter of the ReAL model. The *Re* parameter is assumed to reflect a task simplification that facilitates responses in the compatible block of IAT trials, but cannot be applied to responses in the incompatible block of trials. Consequently, we manipulated whether participants completed an IAT that consisted of either the traditional, multi-block design or a single-block design [38]. The single-block IAT removes the typical multi-block structure of the IAT, such that the compatibility of response assignments varies randomly from trial to trial. Because the response key assignment on one trial does not predict the response key assignment on the next trial, participants are less able to implement a stable and efficient recoding strategy throughout the task for compatible response assignment. Based on the findings of Meissner and Rothermund [3], we expected that the *Re* parameter should be reduced in single-block IATs relative to multi-block IATs. Data, experimental code, and stimuli for all experiments reported in this manuscript are available in the S1 File.

Meissner and Rothermund [3] Experiment 3 also included a recoding-free IAT [23]. However, they found no differences in performance between the single-block and recoding-free IAT variants. Consequently, for efficiency we only use the single-block IAT because it shares more procedural features (e.g., response-stimulus intervals) with the standard IAT than does the recoding-free IAT.

#### Participants

Ninety-four participants from Albert-Ludwigs-Universität Freiburg completed the experiment, *M*_age_ = 21.86, *SD*_age_ = 2.80, 71.28% female. One participant’s data did not write properly, leaving a total sample of 93 participants.

#### Ethics statement

All studies were carried out in strict accordance with the ethical principles as formulated in the WMA Declaration of Helsinki. If research objectives do not involve issues regulated by law (e.g., the German Medicine Act [Arzneimittelgesetz, AMG], the Medical Devices Act [Medizinproduktegesetz, MGP], the Stem Cell Research Act [Stammzellenforschungsgesetz, StFG] or the Medical Association’s Professional Code of Conduct [Berufsordnung der Ärzte]), then no ethics approval is required for social science research in Germany. Our studies had no such objectives, and therefore, no IRB approval or waiver of permission was sought for these studies.

Participation in all studies was entirely voluntary. All participants gave their written informed consent beforehand to participate in the study. They were informed of the study procedures, of their anonymity, and they were told that they were free to leave the study at any point if they wished to. They were naive regarding the purpose of the experiment and were debriefed about the experimental hypotheses at the end of the experiment. These procedures were in accordance with the German Society for Psychology’s research standards (Grundsätze der Forschung am Menschen, C.III, para. 6).

#### Procedure and materials

This experiment consisted of a 2 (IAT procedure: standard; ReAL) x 2 (IAT structure: multi-block; single-block) design, with IAT procedure as a within-participants factor, and IAT structure as a between-participants factor. Participants were tested individually. After completing a demographics questionnaire, participants were randomly assigned to an experimental condition. One experimental session lasted approximately 20 minutes. After completing the experiment, participants were compensated with research credit, chocolate, or 3.50 €.

All IATs were comprised of the same words reflecting four categories: flowers, insects, pleasant, unpleasant (taken from [28]). Stimuli were presented on a black screen, with flower and insect words presented in blue, and pleasant and unpleasant words presented in green. All stimuli were shown in normal German spelling, with the first letter in uppercase and the rest of the word in lowercase letters. Participants completed the IATs by pressing the left or right response keys with their index finger. The experiment was programmed in PsychoPy2 [39].

Participants in the multi-block IAT structure condition completed a standard IAT followed by a ReAL IAT, both with regular blocked formats that include separate blocks for compatible (i.e., flower/pleasant, insect/unpleasant) and incompatible (i.e., insect/pleasant, flower/unpleasant) trials. Participants in the single-block IAT structure condition completed a standard IAT followed by a ReAL IAT, both with compatible and incompatible trials intermixed within blocks for each task [38]. We implemented this fixed presentation order of IATs, rather than counterbalancing the order of standard and ReAL IATs, because the primary focus of this experiment was to validate the ReAL model on the standard IAT. After completing the last block of the second IAT, participants were debriefed and thanked for their participation.

#### IAT structures

**Fig 2:**
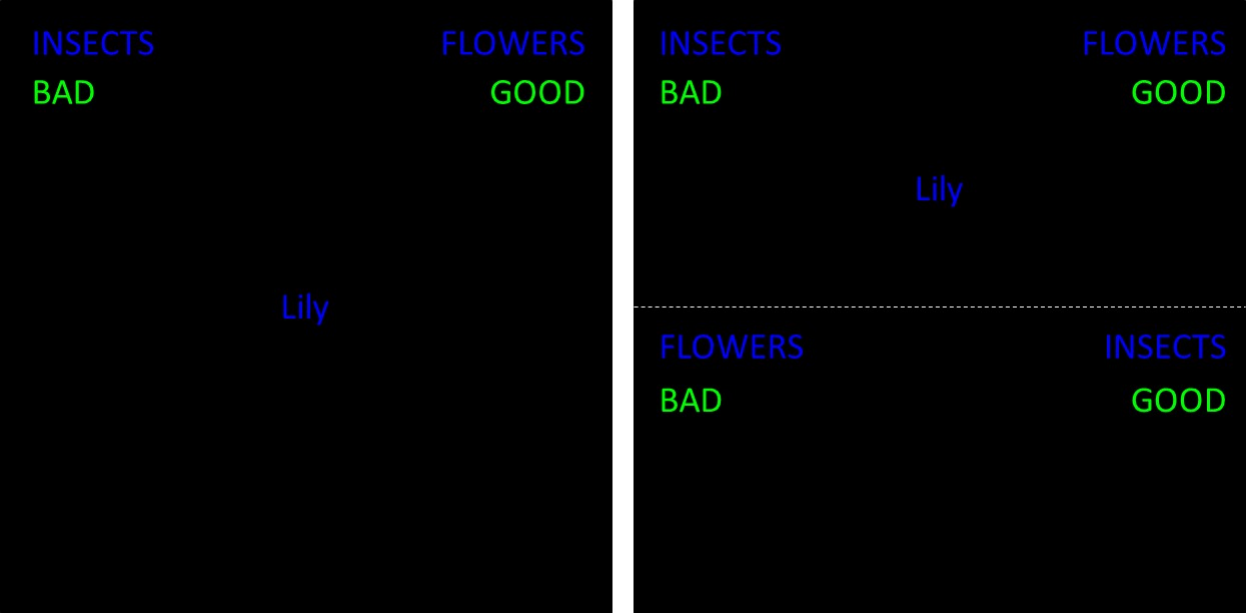
**Example trials on a multi-block IAT (left panel) and a single-block IAT (right panel).** Original stimuli were presented in German.

*Standard IAT*, *multi-block version*. The multi-block version of the standard IAT corresponds to the IAT paradigm described by [13], which is what we call the ‘standard IAT procedure’ throughout this manuscript. Details of the standard IAT procedure are described in Table 1, and the left panel of Fig 2 depicts an example trial of the multi-block IAT configuration. In the first block of 20 trials, participants practiced categorizing attribute stimuli (pleasant, unpleasant), pressing the left key for ‘unpleasant’ and the right key for ‘pleasant’ stimuli. In the second block of 20 trials, participants practiced categorizing target stimuli (flower, insect). Response key assignment was randomized across participants, such that some participants practiced responding to insects with the left key and flowers with the right key (i.e., compatible assignments), and other participants practiced responding to flowers with the left key and insects with the right key (i.e., incompatible assignments).

**Table 1:** Details of the standard and ReAL IAT procedures.

	standard IAT	ReAL IAT
Total number of blocks	7	15
Total number of trials	184	400
Number of critical blocks	4	10
Number of critical trials	120	320
Response deadline	No	Yes
Critical block stimuli presentation	task-switch	task-switch, task-repeat

*Note*: Number of trials does not include one warm-up trial at the beginning of each block.

After completing the practice blocks, participants completed two critical blocks of trials (20, 40 trials, respectively) consisting of all four stimulus types, with the response key assignments fixed throughout both blocks. In the fifth block of 24 trials, participants practiced responding only to target stimuli, with response key assignments reversed relative to their assignment in the previous blocks. Finally, in the sixth and seventh critical blocks (20, 40 trials, respectively), participants responded to all four stimulus types, with the response key assignments fixed and reflecting the reversed key assignments from the fifth block.

During all blocks, the category labels indicating the current response key assignment were shown in the upper left and right corners of the screen for the duration of each block. Every block began with one randomly assigned stimulus as warm-up trial, which was excluded from the analysis [4, 40, 41]. Stimuli were presented randomly, but with the restriction that target and attribute stimuli were presented alternately (i.e., “task-switch”). Stimuli were presented until the correct response was made. When participants made an incorrect response, a red X appeared below the stimulus, and participants had to press the correct response key in order to proceed to the next trial. The next stimulus was presented 200ms after a correct response was made.

*Standard IAT*, *single-block version*. The ways in which the single-block IAT version differs from the mixed-block IAT version are described here. In a single-block IAT, a dashed horizontal line divides the screen into two parts. Stimuli are presented either in the upper or lower part, and the position in which a stimulus appears determines which response mapping the participant uses to respond. Importantly, a single-block IAT does not consist of a single block of trials, per se. Instead, both compatible and incompatible trials appear together within each critical block, and participants complete multiple critical blocks over the course of the task. The right panel of Fig 2 depicts an example trial of the single-block IAT configuration.

In the first two blocks of 32 trials each, participants practiced categorizing target stimuli. When target stimuli appeared in the upper half of the screen, participants responded to flower stimuli with the right key and insect stimuli with the left key. When target stimuli appeared in the lower half of the screen, participants responded to flower stimuli with the left key and insect stimuli with the right key. Participants were randomly assigned to complete either the upper or lower practice assignments in the first block, and then complete the other practice assignments in the second block. In the third block of 32 trials, participants practiced categorizing target stimuli that were randomly presented in both parts of the screen. In the fourth block of 32 trials, participants practiced responding to attribute stimuli. Attribute category labels were the same for the upper and lower parts of the screen, so participants always responded to unpleasant stimuli with the left key, and responded to pleasant stimuli with the right key. Consequently, for the remainder of the task, response key pairings reflected compatible assignments in the upper half of the screen, and incompatible assignments in the lower half of the screen. After completing the practice blocks, participants completed two critical blocks of trials consisting of all four stimulus types. Stimuli randomly appeared in either the top or bottom half of the screen for each trial. Each critical block consisted of 32 compatible trials and 32 incompatible trials, for a total of 128 critical trials. Category labels indicating response key assignments were shown in the top left and right corners of their respective halves of the screen for the duration of each block, such that labels appeared at the top of the screen for stimuli presented in the upper half of the screen, and labels appeared directly below the dashed horizontal line that divided the screen into two parts for stimuli presented in the lower half of the screen.

*ReAL IAT*, *multi-block version*. The multi-block version of the ReAL IAT corresponds to the IAT paradigm described by Meissner and Rothermund [3], which is what we call the ‘ReAL IAT procedure’ throughout this manuscript. Details of the ReAL IAT procedure are described in Table 1. The ways in which the ReAL IAT procedure differs from the standard IAT procedure are described here.

The first (attribute) and second (target) practice blocks consisted of 16 trials each. The third block consisted of 32 practice trials of all four stimulus types, and reflected the response pairings that participants were randomly assigned in the second block. Participants completed compatible versus incompatible blocks in the same order as they did on the standard IAT. The fourth block consisted of 16 practice target trials, with the response key pairings reversed from the second and third blocks. The fifth block consisted of 32 practice trials with all four stimulus types, and reflected the same target response pairings from the fourth block. Blocks six through fifteen were critical blocks of 32 trials each, and blocks alternated between compatible and incompatible response pairings.

For all trials, a response deadline was implemented based on the 75th percentile of each participant’s response latency on the standard IAT. Meissner and Rothermund [3] employed a progressive response deadline procedure, in which all participants started with a 750ms deadline that either increased or decreased across blocks in pre-determined increments depending on response accuracy in previous blocks. However, in subsequent work, Meissner and Rothermund [28, Experiment 2] employed a fixed response deadline of 700ms across all blocks for all participants. In the present research, we combined the advantages of the individualized and standardized approaches (i.e., progressive versus fixed deadlines) by implementing a fixed response deadline based on each participant’s average response latency on the standard IAT. In doing so, response deadlines are set according to each participant’s response tendencies, but remain constant across trials. If participants failed to make a response within this deadline, a red frame appeared around the stimulus. Participants were instructed to respond before the frame appeared, and that they should accept making more mistakes if they need to respond more quickly. Stimuli were presented randomly, but without the task-switch restriction implemented in the standard IAT procedure. Instead, the ReAL IAT procedure consisted of both task-repeat (i.e., target, target; attribute, attribute) and task-switch (i.e., target, attribute; attribute, target) trials, and the two trial types appeared equally often.

*ReAL IAT*, *single-block version*. The ReAL IAT, single-block version was identical to the standard IAT, single-block version with the following exceptions. A response deadline was implemented based on the 75th percentile of each participant’s response latency on the standard IAT, and a red frame appeared around the stimulus if participants failed to make a response within this deadline. Both task-repeat and task-switch trials were presented. Instead of completing two critical blocks of trials, participants completed 10 critical blocks of trials, for a total of 384 critical trials.

#### Results

*Parameter estimation and analysis*. Only responses from critical blocks were included in analyses. Block order (compatible first, incompatible first) was treated as a nuisance variable, and was not included in analyses.

In MPT modeling, the equations for each item category (e.g., flowers, insects, pleasant words, and unpleasant words in both compatible and incompatible block types) predict the proportions of correct versus incorrect responses observed in a given data set. The model’s predictions are compared to participants’ actual data to determine the model’s ability to account for the data, and a goodness-of-fit statistic (e.g., χ^2^, *G*^2^) is computed to reflect the difference between the predicted and observed errors. To date, all research using the ReAL model has estimated model parameters for each individual participant using maximum likelihood estimation until they produced a minimum possible *G*^2^ value, and examined differences between parameters using *t-*tests and ANOVAs.

A primary advantage of estimating parameters at the individual level is that this approach accounts for heterogeneity across participants. However, individual-level estimates can also be relatively noisy: parameter estimation through maximum likelihood depends in part on frequencies of correct and incorrect responses, but participants typically make very few errors, especially on the standard IAT. An alternate estimation approach is to aggregate responses across participants, which provides more stable parameter estimates but ignores heterogeneity across participants.

In the present research, we used a Bayesian approach proposed by [42, 43] to fit a multilevel extension of the ReAL model that treats participants and items as random factors [44] for each model parameter. This approach preserves the benefits of both individual and aggregate estimation methods, in that it allows for participant-level heterogeneity while simultaneously providing a means to aggregate across individuals for stable estimates. This Bayesian method provides two model checks to assess goodness of fit [42]: *T*_1_ summarizes how well the model accounts for the pattern of observed response frequencies aggregated across items and participants within each condition, corresponding to the goodness-of-fit statistic *G*^2^ used in the traditional modeling approach [5]; *T*_2_ summarizes how well the model accounts for the variances and correlations of these frequencies across participants. Furthermore, the Bayesian approach yields highest density intervals (HDIs) for the parameter estimates that can be interpreted like classical confidence intervals. Hypotheses tests for equality between any two parameters can be conducted by checking whether zero is contained in the HDI of the differences between the two parameters.

*The Brief ReAL model*. Thus far, we have only discussed the three primary parameters in the ReAL model: recoding (*Re*), evaluative associations (*A*) and label-based identification of the correct response (*L*). The ReAL model, as validated by Meissner and Rothermund [3] also includes three technical parameters. These technical parameters account for differences resulting from task repetition and task change sequences [19, 23, 45, 46], and also allow *Re* to differ across target versus attribute stimuli. Thus, the ReAL model estimates a total of 10 parameters: one *Re*, two *A* (one for each target category), four *L* (one for each stimulus category), and three technical parameters. The number of parameters a model can estimate depends on the number of non-redundant response categories (i.e., degrees of freedom) that exist in a given task. Because the ReAL IAT procedure includes task-switch and task-repetition trials, it can be parsed into 16 response categories: 4 stimulus types (flower, insect, pleasant, unpleasant) × 2 trial types (compatible, incompatible) × 2 trial sequences (task-switch, task-repetition). These 16 degrees of freedom are sufficient to estimate the 10 parameters of the ReAL model. However, the standard IAT procedure includes only task-switch trials, so it can be parsed into eight response categories. Consequently, the standard IAT procedure does not provide sufficient degrees of freedom to estimate all 10 parameters of the ReAL model–and, thus, the full ReAL model form cannot be validated on the standard IAT procedure.

Here, we present an abbreviated version of the ReAL model that retains the three core parameters (*Re*, *A*, *L*) but does not include the three technical parameters (hereafter referred to as the *Brief ReAL* model). The full set of equations for the Brief ReAL model is listed in the S1 File. The two technical parameters that account for differences resulting from task repetition and task change sequences are obsolete in the context of the standard IAT, which includes only task-switch trials. In the General Discussion we evaluative the validity of omitting the third technical parameter, that allows *Re* to differ across target versus attribute stimuli, and provide a mathematical proof in the supporting information demonstrating that omitting the technical parameters will not change the qualitative nature of the model.

Based on responses to the standard IAT versions (multi-block; single-block), we estimated one *Re* parameter, two *A* parameters (*A*_flower_, *A*_insect_), and four *L* parameters (*L*_flower_, *L*_insect_, *L*_positive_, *L*_negative_) for the Brief ReAL model. All parameters are estimated on a probability scale, ranging [0,1]. Higher values of the *Re* and *L* parameters can be interpreted to reflect greater influence of each cognitive process. In contrast, the *A* parameters are anchored at 0.5, such that values greater than 0.5 indicate positive evaluations of the target, values less than 0.5 indicate negative evaluations of the target, and values not different from 0.5 indicate neutral evaluations of the target. Parameter estimates are listed in Table 2.

**Table 2:** Parameter estimates from Replication Experiment 1.

	standard IAT		ReAL IAT	
	*Multi-block*	*Single-block*	*Multi-block*	*Single-block*
*A*_flower_	0.37 [0.23, 0.51]	0.49 [0.37, 0.60]	0.64 [0.58, 0.69]	0.64 [0.58, 0.69]
*A*_insect_	0.61 [0.48, 0.75]	0.44 [0.31 0.55]	0.40 [0.33, 0.45]	0.38 [0.33, 0.44]
*L*_flower_	0.77 [0.66, 0.85]	0.57 [0.45, 0.67]	0.71 [0.65, 0.76]	0.59 [0.54, 0.65]
*L*_insect_	0.65 [0.50, 0.77]	0.61 [0.49, 0.71]	0.68 [0.62, 0.73]	0.61 [0.55, 0.66]
*L*_positive_	0.89 [0.84, 0.94]	0.85 [0.79, 0.90]	0.87 [0.83, 0.90]	0.88 [0.85, 0.91]
*L*_negative_	0.87 [0.82, 0.92]	0.82 [0.77, 0.88]	0.88 [0.84, 0.91]	0.87 [0.84, 0.91]
*Re*	0.55 [0.36, 0.55]	0.45 [0.28, 0.62]	0.56 [0.45, 0.67]	0.19 [0.02, 0.32]

*Note*: Bracketed values reflect 95% HDIs.

*Model fit*. The Brief ReAL model fit data from the standard IAT versions well, *T*_*1*_ = 12.85, *p* = .63, *T*_*2*_ = 935.20, *p* = .23. However, the ReAL model did not fit data from the ReAL IAT versions well, *T*_*1*_ = 69.20, *p* = .0002, *T*_*2*_ = 7733.00, *p* = .02. The extent to which a multinomial model provides good fit to data is an important first step in demonstrating its’ validity. That said, as is often the case with summary statistics, model fit statistics only tell part of the story. For example, fit statistics depend, in part, on number of observations. In the context of MPT modeling, number of observations is a function of both number of participants and number of trials. Because the ReAL IAT versions consist of more trials than do the standard IAT versions, model fit statistics are not unambiguous evidence that the Brief ReAL model provides better fit than does the ReAL model. Consequently, we include in the S1 File graphs of the observed versus predicted responses frequencies, variances, and covariances for the ReAL and Brief ReAL models. Visual inspection of these graphs indicates that the magnitude of misfit for the ReAL model is small, with observed values rarely falling outside of 95% HDIs for predicted values.

*Standard IAT versions*. Contrary to our predictions that recoding should be reduced by task conditions that minimize the opportunity for participants to implement a stable and efficient recoding strategy, *Re* estimates did not differ between multi-block and single-block IAT variants, 95% HDI of difference [-0.13, 0.30]. On the multi-block IAT, *A* parameters were not in the theoretically-expected direction. Because *A* parameters values greater than 0.5 reflect positive evaluations and values less than 0.5 reflect negative evaluations, we expected the *A* parameter for flowers to be greater than 0.5 and the *A* parameter for insects to be less than 0.5. Instead, on the multi-block IAT, the *A* parameter for flowers was descriptively (though not reliably) less than the *A* parameter for insects, 95% HDI of difference [-0.0004, 0.48], and neither *A* parameter was reliably different from 0.5 (i.e., each parameter’s 95% HDI included 0.5). On the single-block IAT, *A* parameters were in the theoretically-expected direction, such that the *A* parameter for flowers was descriptively greater than the *A* parameter for insects. However, the *A* parameters were not reliably different from one another, 95% HDI of difference [-0.25, 0.14], nor was either parameter reliably different from 0.5. The *L* parameter for flowers was reliably higher in the multi-block versus single-block IAT, 95% HDI of difference [0.06, 0.33], but the other *L* parameters did not differ between IAT variants.

*ReAL IAT versions*. As predicted, *Re* estimates were higher in the multi-block IAT than the single-block IAT, 95% HDI of difference [0.18, 0.58]. Moreover, *A* parameters were reliably different from 0.5 in the theoretically-expected direction, and were reliably different from one another, for both the multi-block IAT, 95% HDI of difference [0.15, 0.33], and for the single-block IAT, 95% HDI of difference [0.16, 0.34]. The *L* parameter for flowers was reliably higher in the multi-block versus single-block IAT, 95% HDI of difference [0.04, 0.19], but the other *L* parameters did not differ between IAT variants.

#### Discussion

The *Re* parameter of the ReAL model reflects a task simplification that influences responses in multi-block IAT versions, but cannot be employed as easily in single-block IAT versions. As predicted, *Re* parameters of the ReAL model were lower for participants who completed single-block versus multi-block versions of the ReAL IAT. However, *Re* parameters from the Brief ReAL model did not differ between single-and multi-block versions for participants who completed the standard IAT. Moreover, the ReAL model produced theoretically-interpretable *A* parameters from both multi- and single-block versions of the ReAL IAT, but the Brief ReAL model did not produce theoretically-interpretable *A* parameters for either version of the standard IAT. And finally, for both the ReAL and Brief ReAL model, *L* parameters for flowers but not other stimuli were higher in multi- than single-block IATs.

### Replication Experiment 2

This experiment conceptually replicated Meissner and Rothermund [3] Experiment 4 to validate the *A* parameters, and consisted of a novel attitude induction paradigm [47]. Participants read a short story about two fictitious soccer teams who competed in a local soccer championship. One of the teams was described negatively, and the other team was described positively. Based on the findings of Meissner and Rothermund [3], we expected that the *A* parameters should reflect the manipulated valence of the novel groups.

#### Participants

Fifty-two participants from Albert-Ludwigs-Universität Freiburg completed the experiment, *M*_age_ = 25.63, *SD*_age_ = 5.01, 59.60% female. Recruitment and compensation procedures were identical to Replication Experiment 1. Because this experiment depends on participants correctly remembering the information presented in the attitude induction task, we applied the same learning exclusion criteria as were used in Meissner and Rothermund [3] Experiment 4. The data of seven participants were excluded because they failed to correctly answer control questions, and 14 participants were excluded because they made more than 25% mistakes in one of the blocks of practice trials. The final sample consisted of 31 participants, *M*_age_ = 25.39, *SD*_age_ = 5.17, 55.00% female.

#### Procedure and materials

This experiment consisted of a 2 (IAT procedure: standard; ReAL) x 2 (team description: Blauheim positive, Rundstedt positive) design, with IAT procedure as a within-participants factor, and team description as a between-participants factor. After completing a demographics questionnaire, participants were randomly assigned to an experimental condition.

*Attitude induction*. Before starting the attitude induction task, participants were instructed to read the following information carefully because they will need to correctly answer questions about it in order to participate in the rest of the experiment. The attitude induction material was formatted like a newspaper article, reporting a match between two soccer teams competing in a tournament. One team was consistently portrayed in a positive manner: members were described as friendly and helpful young men who are active and popular in the community and always play fair. The other team was consistently portrayed in a negative manner: members were described as brutal and mean hooligans who are notorious for their bad behavior on and off the soccer field. The names of the teams (‘Blauheim’ and ‘Rundstedt’) do not exist in reality but, instead, were made up to resemble typical German sounding villages. The article named four players on each team, and players’ names were selected to be typical for young German males in the participants’ age group. The names of the players on each team was also randomized between participants.

To ensure that participants paid close attention to the attitude induction material, they were informed that they would need to notify the experimenter when they had finished the first part of the experiment, and before proceeding to the next task. Additionally, participants had to correctly answer four questions to ensure that they remembered which team was described positively versus negatively (e.g., ‘Which team has the nickname ‘bone crushers’? a: Rundstedt, b: Blauheim’).

*IATs*. Next, participants completed a standard IAT followed by a ReAL IAT, as described in Table 1. Both IATs were comprised of the same words reflecting four categories: Blauheim team members’ names, Rundstedt team members’ names, pleasant, unpleasant. Team members’ names were presented in blue, and pleasant and unpleasant words presented in green. The two team names functioned as the target category labels. Before starting the standard IAT, participants were reminded of the names of the players on each team and instructed to memorize them. After completing the standard IAT but before starting the ReAL IAT, participants had to correctly answer four more questions to ensure that they remembered which team was described positively versus negatively.

#### Parameter estimation and analysis

Data were reduced in the same way as in Replication Experiment 1. Parameters were estimated and analyzed in the same way as in Replication Experiment 1. Team description (Blauheim positive, Rundstedt positive) was treated as a nuisance variable, and was not included in analyses. Parameter estimates are listed in Table 3.

**Table 3:** Parameter estimates from Replication Experiment 2.

	Standard IAT	ReAL IAT
*A*_positive team_	0.33 [0.20, 0.49]	0.55 [0.49, 0.60]
*A*_negative team_	0.67 [0.54, 0.81]	0.43 [0.39, 0.49]
*L*_positive team_	0.54 [0.28, 0.73]	0.71 [0.62, 0.80]
*L*_negative team_	0.50 [0.24, 0.72]	0.69 [0.61, 0.76]
*L*_positive attributes_	0.93 [0.88, 0.98]	0.90 [0.86, 0.94]
*L*_negative attributes_	0.90 [0.83, 0.95]	0.92 [0.88, 0.95]
*Re*	0.56 [0.36, 0.76]	0.58 [0.40, 0.73]

*Note*: Bracketed values reflect 95% HDIs.

*Model fit*. The Brief ReAL model fit data from the standard IAT well, *T*_*1*_ = 7.40, *p* = .50, *T*_*2*_ = 372.00, *p* = .39. The ReAL model provided mixed fit to data from the ReAL IAT, with poor fit for response frequencies, *T*_*1*_ = 32.63, *p* = .01, but good fit for variances and covariances, *T*_*2*_ = 3064.00, *p* = .25. However, visual inspection of graphs of the observed versus predicted responses frequencies, variances, and covariances (https://osf.io/vxyrh/) indicates that the magnitude of misfit is small.

*Standard IAT*. Because *A* parameters values greater than 0.5 reflect positive evaluations and values less than 0.5 reflect negative evaluations, we expected *A* parameters for the team described positively to be greater than 0.5 and *A* parameters for the team described negatively to be less than 0.5. However, the *A* parameter for the team described positively was reliably less than 0.5, the *A* parameter for the team described negatively was reliably greater than 0.5, and the two *A* parameters were reliably different from one another, 95% HDI of difference [-0.59, -0.11].

*ReAL IAT*. Both *A* parameters were in the theoretically-expected direction, with the *A* parameter for the team described positively descriptively (though not reliably) greater than 0.5, the *A* parameter for the team described negatively reliably less than 0.5, and the two *A* parameters reliably different from one another, 95% HDI of difference [0.03, 0.20].

#### Discussion

The *A* parameters of the ReAL model reflect evaluations of the target categories in the IAT. As predicted, *A* parameters were lower for the team that was described negatively than for the team that was described positively, but only for participants’ responses to the ReAL IAT. *A* parameters were in the opposite direction for participants’ responses to the standard IAT.

### Interim discussion

Until now, our goal had been to validate the Brief ReAL model on the standard IAT procedure by replicating the validation studies of Meissner and Rothermund [3]. In both replication experiments, the Brief ReAL model provided good fit to data from standard IAT variants. However, model fit is only the first step in demonstrating validity. A valid model must also produce theoretically-interpretable parameters, but in both replication experiments the parameters of the Brief ReAL model were not sensitive to experimental manipulations: in Replication Experiment 1 recoding parameters did not vary as a function of IAT structure designed to minimize the influence of recoding, and in Replication Experiment 2 association parameters varied in the opposite direction of the evaluative information about the two target groups.

That said, neither of these replication experiments reflect manipulation failures: in both cases the ReAL model replicated the findings of Meissner and Rothermund [3] on the ReAL IAT. Indeed, because participants always completed a standard IAT variant before a ReAL IAT variant, the results so far would seem to suggest that the ReAL model is robust against IAT practice effects. Because the Brief ReAL model and the ReAL model are qualitatively equivalent (see S1 File), the most likely explanation for these differing patterns of results is differences in task procedures between the standard IAT and the ReAL IAT.

## Experiment 3

The ReAL model was developed and validated on a modified IAT procedure that imposes short response deadlines and includes more than double the overall number of trials than does the standard IAT, and also imposes a constraint on the order in which trial categories can be repeated (e.g., two flower stimuli presented consecutively) versus switched (e.g., a flower stimulus followed by an unpleasant stimulus). In previous research [3, 6, 7, 28, 29], as well as in the present research, the ReAL model has performed well and estimated theoretically-interpretable parameters from this modified IAT procedure. However, a qualitatively-equivalent version of the ReAL model (i.e., the Brief ReAL model) did not perform well, and estimated theoretically-uninterpretable parameters from the standard IAT procedure.

Consequently, in Experiment 3, we employed a pre-registered and fully-factorial design to manipulate all of the task procedures that vary between the standard IAT and the ReAL IAT–response deadline, number of trials, trial constraints–to determine the conditions under which the Brief ReAL model can be validly applied to IAT data. Participants in Experiment 3 completed an IAT with flowers and insects as target categories. Because the *A* parameters are conceptualized to reflect target evaluations, their validity will be demonstrated if they are in the theoretically-expected directions: the most basic criterion is that the *A* parameter is descriptively greater for flowers than for insects, and more stringent criteria are that the *A* parameters for flowers are reliably greater than 0.5, the *A* parameters for insects are reliably less than 0.5, and the *A* parameters for flowers and insects are reliably different from one another. Because the *L* parameters are conceptualized to reflect resource-dependent control processes, their validity will be demonstrated if they are reduced in conditions that include versus do not include a response deadline (i.e., conditions that constrain opportunity for *L* to influence responses), as well as if they are reduced in conditions with more relative to fewer trials (i.e., conditions that deplete cognitive resources). The hypothesis that *L* will vary according to number of trials was not included in the preregistration, but the *L* parameter’s sensitivity to number of trials was reported in Meissner and Rothermund [3]. In order to investigate the validity of the *Re* parameters, we additionally manipulated multi- versus single-block IAT structure in the same manner as in Replication Experiment 1. Because the *Re* parameter is conceptualized to reflect a task-simplification process, its validity will be demonstrated if it is reduced in single- versus multi-block conditions (i.e., conditions that disrupt participants from implementing a stable recoding strategy). Hypotheses, experimental design, exclusion criteria, and analysis plan are pre-registered at: https://osf.io/vxyrh/.

### Participants

Based on the average per-cell sample size reported in Meissner and Rothermund [3], we aimed to recruit 40 participants per cell. A total of 621 participants from Albert-Ludwigs-Universität Freiburg and Friedrich-Schiller-Universität Jena completed the experiment, *M*_age_ = 23.65, *SD*_age_ = 4.14, 62.5% female. Recruitment and exclusion procedures were identical to Replication Experiment 1, and participants were paid between 1.50–2 €. The data of 30 participants were excluded because they made more than 25% mistakes on the IAT. The final sample consisted of 591 participants (*M*_age_ = 23.69, *SD*_age_ = 4.18, 63.5% female).

### Procedure and materials

This experiment consisted of a 2 (response deadline: no; yes) x 2 (number of trials: 120; 320) x 2 (trial constraint: switch-only; switch-repeat) x 2 (block structure: multi-block; single-block) fully between-participants design. After completing a demographics questionnaire, participants were randomly assigned to an experimental condition. One experimental session lasted approximately 15 minutes. All IATs were comprised of the same stimuli reflecting flowers, insects, pleasant, and unpleasant concepts as were used in Replication Experiment 1.

#### Response deadline

In Replication Experiments 1 and 2, each participant completed a ReAL IAT variant with a response deadline based on the 75th percentile of their own response latency on the standard IAT. Implementing a response deadline in this manner was possible because participants always completed a standard IAT variant first, followed by a ReAL IAT variant. However, in Experiment 3 participants completed only one IAT variant. Consequently, participants in the response deadline experimental conditions completed an IAT with a 750ms response deadline.

#### Number of trials

Participants in experimental conditions with the standard number of trials completed an IAT with 120 critical trials. Participants in experimental conditions with the ReAL number of trial completed an IAT with 320 critical trials.

#### Trial constraint

Participants in experimental conditions with switch-only trial constraints completed an IAT in which target and attribute stimuli were presented alternately in critical blocks. Participants in experimental conditions with switch-repeat trial constraints completed an IAT in which both task-repeat (i.e., target, target; attribute, attribute) and task-switch (i.e., target, attribute; attribute, target) trials appeared equally often in critical blocks.

#### Block structure

Participants in multi-block experimental conditions completed an IAT reflecting the standard IAT block order depicted in Table 1, in which compatible trials were presented in separate critical blocks from incompatible trials. Participants in single-block experimental conditions completed an IAT in which compatible and incompatible trials were intermixed within critical blocks [38].

### Results

#### Parameter estimation and analyses

Data were reduced, and parameters were estimated and analyzed, in the same way as in Replication Experiment 1. We applied the Brief ReAL models to data from switch-only conditions, and the ReAL model to data from switch-and-repeat conditions. Fit indices for each condition are presented in Table 4. The Brief ReAL model fit data very well from IATs with switch-only trials. The ReAL model fit data well from IATs with 160 switch-and-repeat trials, but indicated misfit for each of the 320-trial IAT variants. However, visual inspection of graphs of the observed versus predicted responses frequencies, variances, and covariances (https://osf.io/vxyrh/) indicates that the magnitude of misfit is small.

**Table 4:** Model fit.

**Brief ReAL model**						
*Response deadline*	*Number of trials*	*Block structure*	*T*_*1*_	*P*	*T*_*2*_	*p*
No	160	multi	30.04	0.50	1083.07	0.54
No	160	single	26.13	0.61	2819.84	0.26
No	320	multi	36.46	0.30	5225.20	0.28
No	320	single	30.11	0.43	11956.00	0.08
yes	160	multi	35.75	0.31	2664.06	0.08
yes	160	single	28.80	0.53	3308.21	0.27
yes	320	multi	39.60	0.16	9413.88	0.43
yes	320	single	40.20	0.16	13084.60	0.12
**ReAL Model**						
*Response deadline*	*Number of trials*	*Block structure*	*T*_*1*_	*p*	*T*_*2*_	*p*
No	160	multi	77.04	0.21	1222.63	0.64
No	160	single	63.58	0.41	2549.64	0.46
No	320	multi	91.46	0.02	9172.44	0.21
No	320	single	76.64	0.09	20490.00	0.01
yes	160	multi	58.51	0.55	2911.34	0.55
yes	160	single	61.13	0.43	4057.19	0.69
yes	320	multi	96.59	0.01	12568.30	0.23
yes	320	single	91.69	0.01	16554.50	0.33

To test the effects of our experimental manipulations, we conducted a series of planned contrasts by subtracting the averaged parameter values from one set of conditions from the averaged values of the corresponding parameters from the other set of conditions for each sample of the posterior distribution. In the section below, we summarize and discuss only the theoretically-predicted comparisons, and in the tables we report all parameter estimates for each experimental condition.

*Response deadline*. To test the effects of response deadline on the parameters of the ReAL and Brief ReAL models, we subtracted the averaged parameter values from conditions with no response deadlines from the averaged values from the corresponding parameters from conditions with response deadlines (Table 5). As predicted, for both models all of the *L* parameters were reliably higher in each experimental condition with no response deadline than in the corresponding condition with response deadline. Additionally, for both models the average of each *L* parameter was reliably higher in experimental conditions without response deadlines compared to conditions with response deadlines. The *L* parameters are conceptualized to reflect resource-dependent control processes, and the influence of such processes should be reduced by response deadlines. Because *L* parameters varied as predicted as a function of response deadline, this pattern of results supports their validity in both models.

**Table 5:** Mean parameter estimates from IATs with versus without response deadlines.

**Brief ReAL Model**					
	*Number of trials*	*Block structure*	*Response deadline*	*No response deadline*	*95% HDI of difference*
*A*_flower_	160	multi	0.44	0.40	[-0.22, 0.13]
	160	single	0.57	0.56	[-0.12, 0.11]
	300	multi	0.54	0.46	[-0.19, 0.02]
	300	single	0.57	0.57	[-0.10, 0.10]
*Mean*			**0.53**	**0.50**	**[-0.03, 0.10]**
*A*_insect_	160	multi	0.58	0.50	[-0.24, 0.11]
	160	single	0.45	0.49	[-0.09, 0.18]
	300	multi	0.48	0.50	[-0.09, 0.13]
	300	single	0.39	0.44	[-0.05, 0.14]
*Mean*			**0.47**	**0.48**	**[-0.08, 0.06]**
*L*_flower_	160	multi	0.37	0.73	[0.16, 0.53]
	160	single	0.19	0.57	[0.24, 0.51]
	300	multi	0.33	0.71	[0.28, 0.48]
	300	single	0.19	0.60	[0.27, 0.56]
*Mean*			**0.27**	**0.65**	**[-0.45, -0.31]**
*L*_insect_	160	multi	0.29	0.69	[0.19, 0.58]
	160	single	0.16	0.52	[0.19, 0.53]
	300	multi	0.22	0.68	[0.33, 0.57]
	300	single	0.16	0.63	[0.31, 0.60]
*Mean*			**0.21**	**0.63**	**[-0.50, -0.34]**
*L*_positive_	160	multi	0.52	0.90	[0.26, 0.48]
	160	single	0.56	0.76	[0.06, 0.37]
	300	multi	0.61	0.88	[0.17, 0.37]
	300	single	0.59	0.83	[0.11, 0.34]
*Mean*			**0.57**	**0.84**	**[-0.33, -0.21]**
*L*_negative_	160	multi	0.49	0.86	[0.23, 0.51]
	160	single	0.41	0.77	[0.19, 0.52]
	300	multi	0.51	0.88	[0.25, 0.49]
	300	single	0.51	0.79	[0.16, 0.41]
*Mean*			**0.48**	**0.82**	**[-0.41, -0.28]**
*Re*	160	multi	0.59	0.61	[-0.22, 0.22]
	160	single	0.20	0.38	[-0.02, 0.38]
	300	multi	0.41	0.44	[-0.14, 0.19]
	300	single	0.10	0.22	[-0.01, 0.26]
*Mean*			**0.33**	**0.41**	**[-0.17, 0.01]**
**ReAL Model**					
	*Number of trials*	*Block structure*	*Response deadline*	*No response deadline*	*95% HDI of difference*
*A*_flower_	160	multi	0.37	0.52	[-0.02, 0.31]
	160	single	0.53	0.61	[-0.03, 0.17]
	300	multi	0.56	0.55	[-0.12, 0.07]
	300	single	0.59	0.56	[-0.13, 0.07]
*Mean*			**0.51**	**0.56**	**[-0.10, 0.01]**
*A*_insect_	160	multi	0.51	0.42	[-0.23, 0.07]
	160	single	0.45	0.46	[-0.08, 0.10]
	300	multi	0.39	0.42	[-0.06, 0.11]
	300	single	0.37	0.43	[-0.03, 0.14]
*Mean*			**0.43**	**0.43**	**[-0.06, 0.05]**
*L*_flower_	160	multi	0.57	0.85	[0.14, 0.41]
	160	single	0.20	0.67	[0.33, 0.60]
	300	multi	0.65	0.80	[0.07, 0.22]
	300	single	0.31	0.68	[0.25, 0.48]
*Mean*			**0.43**	**0.75**	**[-0.37, -0.25]**
*L*_insect_	160	multi	0.67	0.86	[0.07, 0.31]
	160	single	0.21	0.66	[0.31, 0.57]
	300	multi	0.64	0.83	[0.10, 0.27]
	300	single	0.26	0.68	[0.28, 0.55]
*Mean*			**0.44**	**0.75**	**[-0.37, -0.25]**
*L*_positive_	160	multi	0.77	0.91	[0.05, 0.22]
	160	single	0.65	0.89	[0.12, 0.36]
	300	multi	0.74	0.91	[0.10, 0.24]
	300	single	0.68	0.87	[0.09, 0.29]
*Mean*			**0.71**	**0.89**	**[-0.23, -0.14]**
*L*_negative_	160	multi	0.72	0.90	[0.08, 0.28]
	160	single	0.60	0.83	[0.06, 0.39]
	300	multi	0.75	0.89	[0.07, 0.22]
	300	single	0.69	0.85	[0.05, 0.26]
*Mean*			**0.69**	**0.87**	**[-0.23, -0.12]**
*Re*	160	multi	0.56	0.57	[-0.26, 0.27]
	160	single	0.16	0.43	[0.04, 0.48]
	300	multi	0.53	0.57	[-0.14, 0.21]
	300	single	0.18	0.27	[-0.06, 0.25]
*Mean*			**0.36**	**0.46**	**[-0.20, 0.002]**

*Block structure*. To test the effects of block structure on the parameters of the ReAL and Brief ReAL models, we subtracted the averaged parameter values from conditions with multi-block IATs from the averaged values of the corresponding parameters from conditions with single-block IATs (Table 6). As predicted, for both models *Re* parameters were descriptively higher in multi-block conditions than single-block conditions. For both models, this difference was reliably different from zero in all conditions except for in the 160-trial no response deadline conditions. Additionally, for both models the average of each *Re* parameter was reliably higher in multi- versus single-block experimental conditions. The *Re* parameter is conceptualized to reflect recoding as a task-simplification process, and such a process should be reduced by conditions that disrupt the use of a stable recoding strategy. Because *Re* parameters varied as predicted as a function of block structure, this pattern of results supports their validity in both models.

**Table 6:** Mean parameter estimates from multi-block versus single-block IATs.

**Brief ReAL Model**					
	*Response deadline*	*Number of trials*	*Multi-block*	*Single-block*	*95% HDI of difference*
*A*_flower_	No	160	0.40	0.56	[-0.34, 0.02]
	No	300	0.46	0.57	[-0.22, 0.003]
	Yes	160	0.44	0.57	[-0.24, -0.01]
	Yes	300	0.54	0.57	[-0.13, 0.06]
*Mean*			**0.46**	**0.57**	**[0.04, 0.17]**
*A*_insect_	No	160	0.50	0.49	[-0.17, 0.20]
	No	300	0.50	0.44	[-0.05, 0.19]
	Yes	160	0.58	0.45	[0.02, 0.25]
	Yes	300	0.48	0.39	[0.01, 0.18]
*Mean*			**0.51**	**0.44**	**[-0.14, -0.01]**
*L*_flower_	No	160	0.73	0.57	[-0.03, 0.32]
	No	300	0.71	0.60	[-0.02, 0.24]
	Yes	160	0.37	0.19	[0.03, 0.32]
	Yes	300	0.33	0.19	[0.02, 0.25]
*Mean*			**0.53**	**0.39**	**[-0.21, -0.07]**
*L*_insect_	No	160	0.69	0.52	[-0.05, 0.37]
	No	300	0.68	0.63	[-0.09, 0.19]
	Yes	160	0.29	0.16	[-0.03, 0.28]
	Yes	300	0.22	0.16	[-0.07, 0.17]
*Mean*			**0.47**	**0.37**	**[-0.18, -0.02]**
*L*_positive_	No	160	0.90	0.76	[0.01, 0.26]
	No	300	0.88	0.83	[-0.02, 0.14]
	Yes	160	0.52	0.56	[-0.18, 0.11]
	Yes	300	0.61	0.59	[-0.11, 0.13]
*Mean*			**0.73**	**0.68**	**[-0.10, 0.02]**
*L*_negative_	No	160	0.86	0.77	[-0.02, 0.21]
	No	300	0.88	0.79	[0.01, 0.17]
	Yes	160	0.49	0.41	[-0.11, 0.25]
	Yes	300	0.51	0.51	[-0.15, 0.15]
*Mean*			**0.68**	**0.62**	**[-0.13, 0.002]**
*Re*	No	160	0.61	0.38	[-0.03, 0.47]
	No	300	0.44	0.22	[0.04, 0.38]
	Yes	160	0.59	0.20	[0.24, 0.54]
	Yes	300	0.41	0.10	[0.19, 0.42]
*Mean*			**0.51**	**0.23**	**[-0.37, -0.20]**
**ReAL Model**					
	*Response deadline*	*Number of trials*	*Multi-block*	*Single-block*	*95% HDI of difference*
*A*_flower_	no	160	0.52	0.61	[-0.25, 0.08]
	no	300	0.55	0.56	[-0.14, 0.10]
	yes	160	0.37	0.53	[-0.25, -0.06]
	yes	300	0.56	0.59	[-0.10, 0.04]
*Mean*			**0.50**	**0.57**	**[0.02, 0.13]**
*A*_insect_	no	160	0.42	0.46	[-0.19, 0.12]
	no	300	0.42	0.43	[-0.12, 0.09]
	yes	160	0.51	0.45	[-0.02, 0.15]
	yes	300	0.39	0.37	[-0.05, 0.08]
*Mean*			**0.44**	**0.43**	**[-0.06, 0.05]**
*L*_flower_	no	160	0.85	0.67	[0.06, 0.29]
	no	300	0.80	0.68	[0.02, 0.23]
	yes	160	0.57	0.20	[0.21, 0.52]
	yes	300	0.65	0.31	[0.26, 0.44]
*Mean*			**0.72**	**0.46**	**[-0.31, -0.20]**
*L*_insect_	no	160	0.86	0.66	[0.10, 0.32]
	no	300	0.83	0.68	[0.04, 0.27]
	yes	160	0.67	0.21	[0.31, 0.59]
	yes	300	0.64	0.26	[0.27, 0.48]
*Mean*			**0.75**	**0.45**	**[-0.36, -0.24]**
*L*_positive_	no	160	0.91	0.89	[-0.06, 0.09]
	no	300	0.91	0.87	[-0.02, 0.11]
	yes	160	0.77	0.65	[-0.004, 0.25]
	yes	300	0.74	0.68	[-0.04, 0.17]
*Mean*			**0.83**	**0.77**	**[-0.11, -0.01]**
*L*_negative_	no	160	0.90	0.83	[-0.02, 0.15]
	no	300	0.89	0.85	[-0.02, 0.12]
	yes	160	0.72	0.60	[-0.05, 0.29]
	yes	300	0.75	0.69	[-0.04, 0.17]
*Mean*			**0.81**	**0.74**	**[-0.13, -0.02]**
*Re*	no	160	0.57	0.43	[-0.15, 0.43]
	no	300	0.57	0.27	[0.06, 0.48]
	yes	160	0.56	0.16	[0.20, 0.56]
	yes	300	0.53	0.18	[0.22, 0.46]
*Mean*			**0.55**	**0.26**	**[-0.39, -0.18]**

*Number of trials*. To test the effects of number of trials on the parameters of the ReAL and Brief ReAL models, we subtracted the averaged parameter values from the conditions with 160 trials from the averaged values of the corresponding parameters from the conditions with 320 trials (Table 7). Unexpectedly, for both models none of the *L* parameters were reliably different between experimental conditions with 160 versus 320 trials. Similarly, for both models the average of each *L* parameter did not differ between 160 versus 320 trial experimental conditions. The *L* parameters are conceptualized to reflect resource-dependent control processes, and the influence of such processes should be reduced by task conditions that require relatively more cognitive resource. Because *L* parameters did not vary as predicted as a function of number of trials, this pattern of results challenges their validity in both models.

**Table 7:** Mean parameter estimates from 160-trial versus 320-trial IATs.

**Brief ReAL Model**					
	*Response deadline*	*Block structure*	*160 trials*	*320 trials*	*95% HDI of difference*
*A*_flower_	no	multi	0.40	0.46	[-0.12, 0.23]
	no	single	0.56	0.57	[-0.10, 0.14]
	yes	multi	0.44	0.54	[-0.01, 0.21]
	yes	single	0.57	0.57	[-0.09, 0.10]
*Mean*			**0.49**	**0.54**	**[-0.02, 0.11]**
*A*_insect_	no	multi	0.50	0.50	[-0.18, 0.17]
	no	single	0.49	0.44	[-0.19, 0.08]
	yes	multi	0.58	0.48	[-0.20, 0.01]
	yes	single	0.45	0.39	[-0.14, 0.03]
*Mean*			**0.50**	**0.45**	**[-0.12, 0.01]**
*L*_flower_	no	multi	0.73	0.71	[-0.16, 0.15]
	no	single	0.57	0.60	[-0.12, 0.18]
	yes	multi	0.37	0.33	[-0.19, 0.09]
	yes	single	0.19	0.19	[-0.12, 0.12]
*Mean*			**0.46**	**0.46**	**[-0.08, 0.06]**
*L*_insect_	no	multi	0.69	0.68	[-0.17, 0.16]
	no	single	0.52	0.63	[-0.09, 0.31]
	yes	multi	0.29	0.22	[-0.23, 0.08]
	yes	single	0.16	0.16	[-0.11, 0.11]
*Mean*			**0.41**	**0.42**	**[-0.07, 0.09]**
*L*_positive_	no	multi	0.90	0.88	[-0.09, 0.07]
	no	single	0.76	0.83	[-0.06, 0.19]
	yes	multi	0.52	0.61	[-0.03, 0.21]
	yes	single	0.56	0.59	[-0.11, 0.18]
*Mean*			**0.68**	**0.73**	**[-0.02, 0.11]**
*L*_negative_	no	multi	0.86	0.88	[-0.05, 0.10]
	no	single	0.77	0.79	[-0.09, 0.14]
	yes	multi	0.49	0.51	[-0.14, 0.18]
	yes	single	0.41	0.51	[-0.07, 0.27]
*Mean*			**0.63**	**0.67**	**[-0.03, 0.11]**
*Re*	no	multi	0.61	0.44	[-0.39, 0.09]
	no	single	0.38	0.22	[-0.37, 0.05]
	yes	multi	0.59	0.41	[-0.31, -0.04]
	yes	single	0.20	0.10	[-0.23, 0.03]
*Mean*			**0.44**	**0.29**	**[-0.24, -0.05]**
**ReAL Model**					
	*Response deadline*	*Block structure*	*160 trials*	*320 trials*	*95% HDI of difference*
*A*_flower_	no	multi	0.52	0.55	[-0.15, 0.19]
	no	single	0.61	0.56	[-0.16, 0.07]
	yes	multi	0.37	0.56	[0.09, 0.28]
	yes	single	0.53	0.59	[-0.01, 0.14]
*Mean*			**0.51**	**0.56**	**[-0.01, 0.11]**
*A*_insect_	no	multi	0.42	0.42	[-0.15, 0.14]
	no	single	0.46	0.43	[-0.13, 0.09]
	yes	multi	0.51	0.39	[-0.21, -0.03]
	yes	single	0.45	0.37	[-0.13, -0.02]
*Mean*			**0.46**	**0.40**	**[-0.11, -0.002]**
*L*_flower_	no	multi	0.85	0.80	[-0.13, 0.03]
	no	single	0.67	0.68	[-0.13, 0.14]
	yes	multi	0.57	0.65	[-0.04, 0.22]
	yes	single	0.20	0.31	[-0.02, 0.22]
*Mean*			**0.57**	**0.61**	**[-0.02, 0.09]**
*L*_insect_	no	multi	0.86	0.83	[-0.10, 0.04]
	no	single	0.66	0.68	[-0.12, 0.16]
	yes	multi	0.67	0.64	[-0.15, 0.11]
	yes	single	0.21	0.26	[-0.07, 0.17]
*Mean*			**0.60**	**0.60**	**[-0.05, 0.06]**
*L*_positive_	no	multi	0.91	0.91	[-0.05, 0.06]
	no	single	0.89	0.87	[-0.10, 0.06]
	yes	multi	0.77	0.74	[-0.12, 0.06]
	yes	single	0.65	0.68	[-0.10, 0.16]
*Mean*			**0.80**	**0.80**	**[-0.05, 0.04]**
*L*_negative_	no	multi	0.90	0.89	[-0.06, 0.06]
	no	single	0.83	0.85	[-0.08, 0.10]
	yes	multi	0.72	0.75	[-0.08, 0.14]
	yes	single	0.60	0.69	[-0.09, 0.25]
*Mean*			**0.76**	**0.79**	**[-0.02, 0.09]**
*Re*	no	multi	0.57	0.57	[-0.26, 0.26]
	no	single	0.43	0.27	[-0.38, 0.09]
	yes	multi	0.56	0.53	[-0.19, 0.13]
	yes	single	0.16	0.18	[-0.13, 0.15]
*Mean*			**0.43**	**0.39**	**[-0.15, 0.05]**

*Theoretical interpretability of A parameters*. *A* parameters for each experimental condition are reported in Table 8. To test whether the ReAL and Brief ReAL models can produce theoretically-interpretable *A* parameters, we report three sets of tests, each corresponding to increasingly stringent tests of interpretability. As a first test, we observed whether *A* parameters were in the theoretically-expected direction: *A* parameters for flowers should be descriptively greater than *A* parameters for insects. As a second, more stringent test, we observed whether *A* parameters were reliably different from neutral: the lower limit of the 95% HDI for *A* parameters for flowers should not include 0.5 (indicating positive evaluations), and the upper limit of the 95% HDI for *A* parameters for insects should not include 0.5 (indicating negative evaluations). As a third, more stringent test, we investigated whether *A* parameters for flowers were reliably greater than *A* parameters for insects. To do so, we subtracted the posterior distributions of the *A* parameters for insects from the posterior distributions of the *A* parameters for flowers in each condition.

**Table 8:** Mean *A* parameter estimates and 95% HDIs of planned contrasts.

**Brief ReAL Model**							
*Response deadline*	*Number of trials*	*Block structure*	*A*_flower_	*95% HDI of A*_flower_	*A*_insect_	*95% HDI of A*_insect_	*95% HDI of A*_flower_—*A*_insect_
No	160	Multi	0.40	[0.25, 0.55]	0.50	[0.35, 0.65]	[-0.36, 0.14]
No	160	Single	0.56	[0.47, 0.65]	0.49	[0.37, 0.60]	[-0.10, 0.23]
No	320	Multi	0.46	[0.38, 0.54]	0.50	[0.41, 0.59]	[-0.19, 0.11]
No	320	Single	0.57	[0.50, 0.65]	0.44	[0.36, 0.51]	[0.02, 0.26]
Yes	160	Multi	0.44	[0.36, 0.53]	0.58	[0.49, 0.67]	[-0.28, 0.01]
Yes	160	Single	0.57	[0.50, 0.64]	0.45	[0.38, 0.51]	[0.01, 0.23]
Yes	320	Multi	0.54	[0.47, 0.61]	0.48	[0.42, 0.54]	[-0.05, 0.17]
Yes	320	Single	0.57	[0.51, 0.64]	0.39	[0.33, 0.45]	[0.09, 0.28]
**ReAL model**							
*Response deadline*	*Number of trials*	*Block structure*	*A*_flower_	*95% HDI of A*_flower_	*A*_insect_	*95% HDI of A*_insect_	*95% HDI of A*_flower_—*A*_insect_
No	160	Multi	0.52	[0.37, 0.67]	0.42	[0.30, 0.56]	[-0.11, 0.30]
No	160	Single	0.61	[0.52, 0.68]	0.46	[0.37, 0.54]	[0.02, 0.27]
No	320	Multi	0.55	[0.45, 0.63]	0.42	[0.35, 0.49]	[-0.002, 0.24]
No	320	Single	0.56	[0.48, 0.64]	0.43	[0.36, 0.51]	[0.01, 0.25]
Yes	160	Multi	0.37	[0.29, 0.46]	0.51	[0.43, 0.58]	[-0.25, -0.004]
Yes	160	Single	0.53	[0.48, 0.58]	0.45	[0.41, 0.49]	[0.01, 0.15]
Yes	320	Multi	0.56	[0.53, 0.60]	0.39	[0.34, 0.44]	[0.10, 0.24]
Yes	320	Single	0.59	[0.54, 0.65]	0.37	[0.34, 0.41]	[0.14, 0.29]

The Brief ReAL model consistently estimated *A* parameters that were in the theoretically-expected direction for single-block IATs, such that *A* parameters for flowers were descriptively larger than *A* parameters for insects. However, in the context of multi-block IATs, the Brief ReAL model performed much more poorly on this criterion, estimating *A* parameters in the theoretically-expected direction only for the 320 trial response deadline IAT. The Brief ReAL model never met the more stringent criterion of estimating *A* parameters that are reliably different from 0.5. The Brief ReAL model estimated *A* parameters that are reliably different from one another in all single-block IAT conditions except for the 160 trial no response deadline IAT, but never in multi-block IAT conditions. The Brief ReAL model met all three criteria for theoretical interpretability only for the 320-trial response deadline single-block IAT.

The ReAL model consistently estimated *A* parameters that were in the theoretically-expected direction for almost all IATs with switch-and-repeat trials, such that *A* parameters for flowers were descriptively larger than *A* parameters for insects. Only the 160-trial response deadline multi-block IAT differed from this pattern of results, and unexpectedly estimated *A* parameters for flowers that were reliably lower than *A* parameters for insects. The ReAL model met the more stringent criterion of estimating *A* parameters that are reliably different from 0.5 in about half of conditions, and estimated *A* parameters that are reliably different from one another in most conditions. The ReAL model met all three criteria for theoretical interpretability in two conditions: the multi-block and single-block versions of the 320-trial response deadline IAT.

*Interaction analyses*. In addition to the main effects of experimental condition reported above, we also examined the 2- and 3-way interactions between conditions. No interactions were reliably different from zero for the Brief ReAL model, and 10.74% of interactions were reliably different from zero for the ReAL model. Moreover, we examined the main effects and 2-way interactions between IAT counterbalance order and collection city (Freiburg, Jena): 7.14% of these effects were reliably different from zero. We report these analyses in full in the S1 File. Given that the incidence rates of reliable effects are relatively low, and close to a 5% false positive rate, we do not put much weight on them.

## General discussion

The ReAL model [3] quantifies the contribution of three qualitatively distinct processes–recoding, associations, and accuracy-orientation–to responses on the IAT, but has been validated only on a modified version of the IAT procedure. The present research was initially aimed to validate an abbreviated version of the ReAL model on the standard IAT procedure [13], but morphed into a comprehensive investigation into the scope and interpretation of the ReAL model for the IAT.

Our efforts to validate an abbreviated version of the ReAL model on the standard IAT paradigm were generally–but not entirely–unsuccessful. The Brief ReAL model consistently estimated theoretically-interpretable *Re* parameters, and *L* parameters that varied as predicted by response deadline. However, the *L* parameters estimated by the Brief ReAL model did not vary as predicted by number of trials. Moreover, and importantly, the biggest threat to the validity of the Brief ReAL model is its estimation of *A* parameters, which were theoretically consistent only under a narrow range of task conditions. In Replication Experiment 1, the Brief ReAL model estimated *A* parameters that only met the basic validity criterion of directional consistency (i.e., *A* for flowers greater than *A* for insects) for the single-block IAT, and did not meet any validity criteria for the multi-block IAT–nor did it meet any validity criteria in Replication Experiment 2. That said, Experiment 3 revealed more nuance to the task conditions under which the Brief ReAL model estimated valid *A* parameters: it mostly estimated directionally-consistent *A* parameters in the single-block but not multi-block conditions, but met more stringent criteria for validity (i.e., *A* parameters that are reliably different from neutral and reliably different from each other) only in one condition: the 320 trial response deadline single block IAT. This pattern of results suggests that very specific task conditions are necessary for the Brief ReAL model to estimate the most stringently-interpretable parameters.

In contrast, the ReAL model generally performed well. Both replication experiments reproduced the patterns of results observed in Meissner and Rothermund [3]. The ReAL model consistently estimated theoretically-interpretable *Re* parameters, and *L* parameters that varied as predicted by response deadline. Like the Brief ReAL model, the *L* parameters estimated by the ReAL model did not vary as predicted by number of trials (a point we elaborate upon below). However, unlike the Brief ReAL model, the ReAL model estimated *A* parameters that met all validity criteria in Replication Experiments 1 and 2, as well as directionally-consistent *A* parameters in almost all conditions of Experiment 3. That said, in Experiment 3 the ReAL model only met the most stringent criteria for validity in two conditions: the multi- and single-block versions of the 320-trial response deadline IAT. In addition to demonstrating the validity of the ReAL model under different task conditions, the present research also demonstrates the robustness of the ReAL model across different parameter estimation methods. All previous ReAL model research had employed maximum likelihood estimation. In contrast, the present research relied on Bayesean hierarchical parameter estimation, and Replication Experiments 1 and 2 reproduced the patterns of results reported in Meissner and Rothermund [3]. Moreover, the present research suggests that the ReAL model is robust against practice effects because, in both Replication Experiments 1 and 2, participants completed a standard IAT before completing the ReAL IAT. Taken together, the present research provides relatively straightforward evidence for the validity of the ReAL model across a variety of analytic methods, task conditions, and IAT variants–as long as the IAT includes both switch and repeat trials.

### Theoretical implications

The task conditions under which the ReAL and Brief ReAL models performed best provide insight into the theoretical underpinnings of the models. For example, in Experiment 3 the ReAL model and the Brief ReAL model both performed better in single-block IAT conditions than multi-block IAT conditions in terms of consistently estimating theoretically-interpretable parameters. For the ReAL model the difference was slight: *A* parameters followed theoretical expectations in all four single-block IAT conditions and in three of four multi-block IAT conditions. However, for the Brief ReAL model the difference between single- and multi-block IAT variants was stark. The Brief ReAL model consistently estimated *A* parameters that met at least the minimum criterion for theoretical interpretability (i.e., *A* for flowers greater than *A* for insects) in all single-block IAT conditions, and the highest thresholds of interpretability in the 320 trial response deadline single-block IAT. In contrast, Brief ReAL model met the minimum threshold for theoretical interpretability in only one multi-block IAT condition–the 320 trial response deadline single-block IAT–and never met higher thresholds of interpretability. This pattern of results suggests the perhaps puzzling implication that the ReAL model and, especially, the Brief ReAL model are better-suited for single-block than multi-block IAT variants. We discuss potential explanations and implications for this finding below.

#### Conditions that constrain control

The extent to which control processes can influence responses offers one possible explanation for the ReAL and Brief ReAL models’ superior performance in single- versus multi-block IATs. At face value, responses are more difficult to control in single- versus multi-block IATs because the correct response mapping changes for each trial on the single-block IAT, depending on where a target stimulus appears onscreen. Indeed, this difference in task difficulty can be observed by comparing label-based identification parameters–which are conceptualized to reflect the controlled search for the category label to which the stimulus belongs and its’ corresponding response key–estimated from single- versus multi-block IATs. In both Replication Experiment 1 and Experiment 3, label-based identification parameters are lower in single- versus multi-block IAT variants. This pattern of results suggests that block structure moderates the influence of control processes in the IAT.

Of course, block structure is not the only task feature that can affect the influence of control in the IAT. Traditional dual-process models of social cognition generally assume that control processes depend more heavily on the availability of cognitive resources than do other cognitive processes [48, 49]. Single-block IATs would seem to require more cognitive resources than multi-block IATs because participants have to continually hold the stimulus-location rule in working memory. All in all, these findings suggest that the ReAL model performs well under a variety of task conditions that reduce the availability of cognitive resources and, thus, constrain the influence of control.

Not only does the present research help to identify task conditions under which the ReAL and Brief ReAL models perform best, but it also sheds light onto the qualitative nature of cognitive processes reflected in the ReAL model. Label-based identification is conceptualized as a control processes, and control processes are often assumed to possess a specific profile of features: they operate slowly and intentionally, depend on cognitive resources, operate within conscious awareness, and can be stopped once initiated [48, 49]. This profile of features is sometimes assumed to covary perfectly, such that a process that depended on cognitive resources should also operate slowly, etc. However, this assumption conflates operating principles that describe the qualitative nature of a process (e.g., it always produces a correct response) with operating conditions that describe the boundary conditions under which it exerts influence (e.g., it produces responses slowly). Distinguishing between operating principles and operating conditions [50] provides a nuanced perspective on the processes that contribute to responses on implicit measures. Indeed, as Experiment 3 indicates, label-based identification is a relatively slow process, in that it is reduced by response deadline. Additionally, it is a relatively resource-dependent process, in that it is reduced by the more cognitively-taxing single-block IAT structure. However, it is not reduced by completing an extended number of trials. Taken together, this pattern of results provides a nuanced view of the qualitative nature of label-based identification: it is a relatively slow process that to an extent depends on some types of cognitive resources (e.g., the working memory taxed by inconsistent response mappings) but not others (e.g., sustained attention across extended trials).

#### Conditions that facilitate response biases

Structural differences between the single- and multi-block IATs may offer another explanation for the pattern of results that emerged in the present research. As articulated above, single-block IATs are more challenging than multi-block IATs because the correct response mapping changes for each trial, depending on where a target stimulus appears onscreen. However, there is one point of stability in the single-block IAT: the correct response mapping for attribute stimuli stays the same, regardless of where a target stimulus appears. Given this consistency in attribute response mapping, the single-block IAT would seem to be especially susceptible to valence-based response biases. Other MPTs include a parameter that accounts for response biases [17, 51, 52]. Because the ReAL model does not include a response bias parameter, response tendencies induced by the stability of attribute responses mappings would be reflected in association parameters. In turn, this explanation may be why the ReAL and Brief ReAL model variants produced more theoretically-interpretable *A* parameters in single- versus multi-block IATs: the task procedures of the single-block IATs induced greater reliance on valence-based responses, which in turn is reflected in the *A* parameters.

#### Recoding targets versus attributes

We have, thus far, characterized the ReAL and Brief ReAL models as being qualitatively equivalent to one another. While this is true in theory, the model variants are only equivalent when recoding does not vary between target and attribute stimuli, as is demonstrated in the mathematical proof provided in the supplement. The ReAL model allows for the possibility that recoding varies between target and attribute stimuli by way of the technical parameter *attReC*, such that *Re* can be smaller for targets than for attributes. However, the standard IAT does not provide sufficient degrees of freedom to estimate any of the ReAL model’s technical parameters. The standard IAT also does not provide sufficient degrees of freedom to estimate two separate *Re* parameters–one for target stimuli and another for attribute stimuli–which would be conceptually equivalent to estimating one *Re* parameter and one *attReC* parameter. Consequently, the ReAL and Brief ReAL model may not be qualitatively equivalent under conditions in which recoding varies between target and attribute stimuli.

Because the Brief ReAL model includes only one *Re* parameter, it tacitly constrains recoding to be equal across target and attribute stimuli. However, as described in the mathematical proof in the supplement, if recoding for targets is in fact smaller than recoding for attributes, the Brief ReAL model will compensate by changing association parameter estimates. Consider an insightful example suggested by a reviewer: In reality a participant has a moderate positive flower evaluation (*A*_flower_ = 0.55) and a moderate negative insect evaluation (*A*_insect_ = 0.45), but her recoding for targets (*Re*_target_ = 0.30) is less than her recoding for attributes (*Re*_attributes_ = 0.55).Plugging these values into the equations provided in the mathematical proof in the supplement, the Brief ReAL model produces parameter estimates suggesting that the participant has moderate negative flower evaluations (*A’*_flower_ = 0.44) and moderate positive insect evaluations (*A’*_insect_ = 0.56). This outcome mirrors the pattern of theoretically-invalid association parameters reported across experiments in the present research.

To test the possibility that the Brief ReAL model is fundamentally flawed because it includes only one *Re* parameter and, thus, incorrectly constrains recoding to be equal across targets and attributes, we reconfigured the Brief ReAL model and applied it to the data from Experiment 3. Specifically, we gained one degree of freedom by constraining the *L* parameter for flowers to be equal to the *L* parameter for insects, and used this degree of freedom to estimate separate *Re* parameters for targets and attributes. (We chose this constraint, in part, because the present research consistently revealed descriptively small differences between *L* parameters for flowers and insects, which suggests that setting them equal to one another should not severely harm model fit.) If the single *Re* parameter causes the Brief ReAL model to estimate theoretically-invalid association parameters, then this Brief ReAL model variant with separate recoding parameters for targets and attributes should estimate valid associations parameters. However, this was not the case. Instead, the pattern of results for this Brief ReAL model variant largely replicated the pattern of results for the Brief ReAL model as reported in the main text: the Brief ReAL model variant with two *Re* parameters generally estimated *A* parameters in the theoretically-expected directions better for single-block than multi-block IATs, but rarely estimated *A* parameters that met more stringent criteria for theoretical interpretability. The results of these analyses (as well as exploratory analyses on other Brief ReAL model specifications) are reported in full in the S1 File, and indicate that the relatively poor performance of the Brief ReAL does not solely reflect a flawed assumption about recoding.

### Limitations and future directions

The present research is limited in several ways. For example, the IAT is often used to assess attitudes towards people (e.g., political candidates: [53]), groups (e.g., racial majorities and minorities: [54]), and things (e.g., addictive substances: [55]) that have important implications in daily life and throughout society. In contrast, the targets in the IATs used in the present research (i.e., flowers, insects, fictitious soccer teams) are largely inconsequential. From a basic science perspective, our use of ad hoc or relatively simplistic targets helped us to precisely manipulate our constructs of interest without the additional noise that might be associated with more meaningful target stimuli. On the other hand, such noise might actually reflect important motivations (e.g., egalitarianism), more complex evaluations (e.g., ambivalence), or other processes that contribute to consequential real-world outcomes. Previous research has used the ReAL model to assess attitudes in important domains, such as gender [6] and romantic relationships [7]. Future research should build upon that work, along with the present research, in order to better understand the extent to which the theoretical assumptions articulated in the model align with cognitive processes across qualitatively-different domains.

Beyond circumscribing the limits of the application of the ReAL and Brief ReAL models, the present research also provided insight into the qualitative nature of label-based identification: it is slow but somewhat sustained. These findings extend the validation evidence for the parameters of the ReAL model initially reported by Meissner and Rothermund [3]. However, other questions remain about the qualitative nature of the ReAL model’s parameters–perhaps most interestingly, recoding. For example, is recoding a domain-general process, or does it vary according to the attitude object [56]? Are people aware of when they employ recoding as a task-simplification strategy [57], and if so, do they employ it intentionally? MPT models like the ReAL model are especially well-positioned to provide this level of insight into the processes that contribute to implicit social cognition [58–60]. Consequently, further investigation into these and other operating conditions of the cognitive processes reflected in the ReAL model represents a fruitful direction for future research.

## Conclusion

The present research delineates the task conditions under which the ReAL and Brief ReAL models can be validly applied to the IAT. The ReAL model is relatively robust across a variety of analysis methods, task conditions, and IAT procedures–as long as they include both switch and repeat trials–and future research should continue to use it to investigate theoretical and applied questions. In contrast, the Brief ReAL model is only valid under a narrow range of task conditions, and should not be applied to data from the standard IAT paradigm.

MPTs such as the ReAL model can be used as a tool to estimate the influence of associations to responses on implicit measures separately from the influence of other processes. To the extent that associations are of primary interest to researchers who use implicit measures, the present research provides a roadmap to task conditions that can be applied in conjunction with MPTs to optimize the assessment of associations.

## Supporting information

S1 File (TXT)
